# 5'-nucleotidase cN-II emerges as a new predictive biomarker of response to gemcitabine/platinum combination chemotherapy in non-small cell lung cancer

**DOI:** 10.18632/oncotarget.24505

**Published:** 2018-02-16

**Authors:** Francesca Toffalorio, Mariacarmela Santarpia, Davide Radice, Christopher Adrian Jaramillo, Gianluca Spitaleri, Michela Manzotti, Chiara Catania, Lars Petter Jordheim, Giuseppe Pelosi, Godefridus J. Peters, Carmelo Tibaldi, Niccola Funel, Lorenzo Spaggiari, Filippo de Braud, Tommaso De Pas, Elisa Giovannetti

**Affiliations:** ^1^ Medical Oncology Unit of Respiratory Tract and Sarcomas, New Drugs Development Division, European Institute of Oncology, Milan, Italy; ^2^ Medical Affairs, Roche Spa, Monza, Italy; ^3^ Medical Oncology Unit, Department of Human Pathology, University of Messina, Messina, Italy; ^4^ Epidemiology and Biostatistics Division, European Institute of Oncology, Milan, Italy; ^5^ Department of Medical Oncology, VU University Medical Center, Amsterdam, The Netherlands; ^6^ Thoracic Oncology Division, European Institute of Oncology, Milan, Italy; ^7^ Division of Pathology and Laboratory Medicine, European Institute of Oncology, Milan, Italy; ^8^ Centre de Recherche en Cancérologie de Lyon, INSERM 1052/CNRS UMR 5286, Lyon, France; ^9^ Department of Oncology and Hemato-Oncology, University of Milan, Milan, Italy; ^10^ Inter-Hospital Pathology Division, Science and Technology Park, IRCCS MultiMedica, Milan, Italy; ^11^ Division of Oncology, Department of Oncology, S. Luca Hospital, Lucca, Italy; ^12^ CNR-Nano, Institute of Nanoscience and Nanotechnology, Pisa, Italy; ^13^ Cancer Pharmacology Laboratory, AIRC Start-Up Unit, University of Pisa, Pisa, Italy; ^14^ Thoracic Surgery Division, European Institute of Oncology, Milan, Italy

**Keywords:** lung cancer, pharmacogenetics, response, gemcitabine, nucleotidase

## Abstract

A number of pharmacogenetic studies have been carried out in non-small-cell lung cancer (NSCLC) to identify and characterize genes involved in chemotherapy activity. However, the results obtained so far are controversial and no reliable biomarker is currently used to predict clinical benefit from platinum-based chemotherapy, which represents the cornerstone of treatment of advanced NSCLC. This study investigated the expression levels of ERCC1 and of six genes (RRM1, RRM2, hENT1, dCK, cN-II and CDA) involved in gemcitabine metabolism in locally/advanced NSCLC patients treated with gemcitabine/platinum combination. Gene expression was assessed by quantitative-PCR in laser-microdissected specimens and correlated with tumor response. Frequency distribution of responses above and below the median expression level of biomarkers was compared using a two-sided Fisher’s test. 5′-nucleotidase (cN-II) was the only gene differently expressed (*p* = 0.016) in the responders (complete/partial-response) compared to non-responders (stable/progressive disease). In the multivariate analysis, overexpression of this catabolic enzyme of gemcitabine remained a significant negative predictive factor. Patients with low cN-II had a modest trend toward increased survival, while both survival and progression-free survival were significantly longer in a more homogenous validation cohort of 40 advanced NSCLC (8.0 vs. 5.1 months, *p* = 0.026). Moreover, *in vitro* studies showed that silencing or pharmacological inhibition of cN-II increased the cytotoxicity of gemcitabine. This is the first study demonstrating the role of cN-II as a predictor of response to gemcitabine/platinum combinations in NSCLC. Its validation in prospective studies may improve clinical outcome of selected patients.

## INTRODUCTION

Over one million people worldwide die every year due to lung cancer, making it the leading cause of cancer-related deaths [1]. Non-small-cell lung cancer (NSCLC) accounts for approximately 85% of all lung cancer cases. Only a minority of patients are diagnosed with localized, early-stage disease for which the optimal treatment remains surgical resection, lobectomy or pneumonectomy, with curative intent, followed by adjuvant chemotherapy, when indicated [2–4]. In most patients NSCLC presents at diagnosis as a locally advanced or metastatic disease. A number of patients with locally advanced disease can undergo surgery following induction chemotherapy, but most cases are definitely treated with chemotherapy, such as the metastatic patients [5]. In recent years, the treatment of metastatic NSCLC patients has been revolutionized by the introduction of new therapeutic agents specifically designed to target somatically activated oncogenes, such as mutant EGFR and rearranged ALK [6–8]. However, only relatively small subgroups of patients, mainly with adenocarcinoma histology, derive benefit from these targeted therapies. Moreover, despite initial remarkable responses to EGFR or ALK tyrosine kinase inhibitors, almost all patients progress due to acquired drug resistance [9]. For those patients without any targetable driver mutations, platinum-based chemotherapy represents the standard of care. However, cytotoxic chemotherapy is burdened by an unsatisfactory response rate, which is less than 30% irrespective of the drug combination regimen administered, and offers only a modest improvement of survival, which still remains poor for these patients. The response to treatment is also characterized by large inter-patient variability. So far, a number of studies have evaluated the predictive value of different biomarkers, and several genetic and epigenetic alterations, including gene mutations, gene amplification, single nucleotide polymorphisms or altered gene/protein expression, have been associated with treatment outcome to chemotherapy in NSCLC [10–13]. However, most data on possible predictors of response are inconclusive suggesting that pharmacogenetic associations may not always be reproducible when focusing on single candidate biomarkers explored in small size series, without standardized unbiased methods, as well as in different settings for tumor type, stage and evaluation of treatment outcome [13, 14]. Further studies integrating different candidate biomarkers in homogeneous populations, with standardized methods and objective parameters for the evaluation of drug activity are urgently warranted.

Most previous biomarkers studies in NSCLC have focused on key enzymes of the Nucleotide Excision Repair (NER) pathway, such as ERCC1, which can remove bulky adducts and intrastrand crosslinks by platinum-compounds and thus modulate response to these cytotoxic drugs [15, 16]. Indeed, ERCC1 expression levels have been variably associated with response to cisplatin-based chemotherapy and survival in patients with NSCLC in several retrospective studies [17, 18].

Fewer studies evaluated molecular determinants of gemcitabine activity. Gemcitabine (difluorodeoxycytidine; dFdC) is a deoxycytidine analogue whose metabolism parallels that of arabinofuranosylcytosine (AraC), but has a distinct mechanism of action [19]. Because of its hydrophilicity, gemcitabine does not cross the membrane by diffusion and it is transported into cells mostly by the human equilibrative nucleoside transporter 1 (hENT1). A deficiency of this transporter has been associated with drug resistance in *in vitro* studies [19, 20] and in pancreatic cancer patients [21]. Following cellular uptake, gemcitabine requires intracellular phosphorylation to produce active diphosphate (dFdCDP) and triphosphate (dFdCTP) forms that act by inhibiting the enzyme ribonucleotide reductase (RR) and DNA synthesis, respectively. Deoxycytidine kinase (dCK) is the rate-limiting enzyme in this biotransformation and its deficiency has been associated with resistance to gemcitabine in NSCLC cells [19] and in tumor xenografts [22] but no association with clinical outcome was observed in patients with NSCLC treated with gemcitabine-based chemotherapy [23].

Some other studies have evidenced an association between disease response and mRNA levels of the ribonucleotide reductase regulatory subunit (RRM1). Ribonucleotide reductase is a key enzyme for DNA synthesis, is involved in DNA repair and gemcitabine metabolism and overexpression of RRM1 was associated with gemcitabine resistance in NSCLC cell lines [24]. In stage III-IV NSCLC patients treated with gemcitabine/cisplatin a high RRM1 expression was related to a poor outcome [17, 25–27].

Cytidine deaminase (CDA) and cytoplasmic 5′-nucleotidase II (cN-II) are considered the major gemcitabine inactivation enzymes. Their crucial role was demonstrated in *in vitro* experiments by modulating their activity with specific inhibitors [28]. Resistance to gemcitabine has been demonstrated in cells overexpressing CDA *in vitro* [19]. However, the role of CDA may be more important in the pharmacokinetics of gemcitabine, since a high systemic CDA level was associated with a poor efficacy and a low CDA levels with increased, sometime lethal toxicity [29]. cN-II levels were significantly lower in patients with chronic lymphocytic B-leukemia responsive to cladribine than in non-responders [30]. cN-II expression has been considered as a new potential target [31], but might also be an independent prognostic factor in patients with NSCLC treated with gemcitabine, with lower levels associated with a poor prognosis [23].

**Figure 1:**
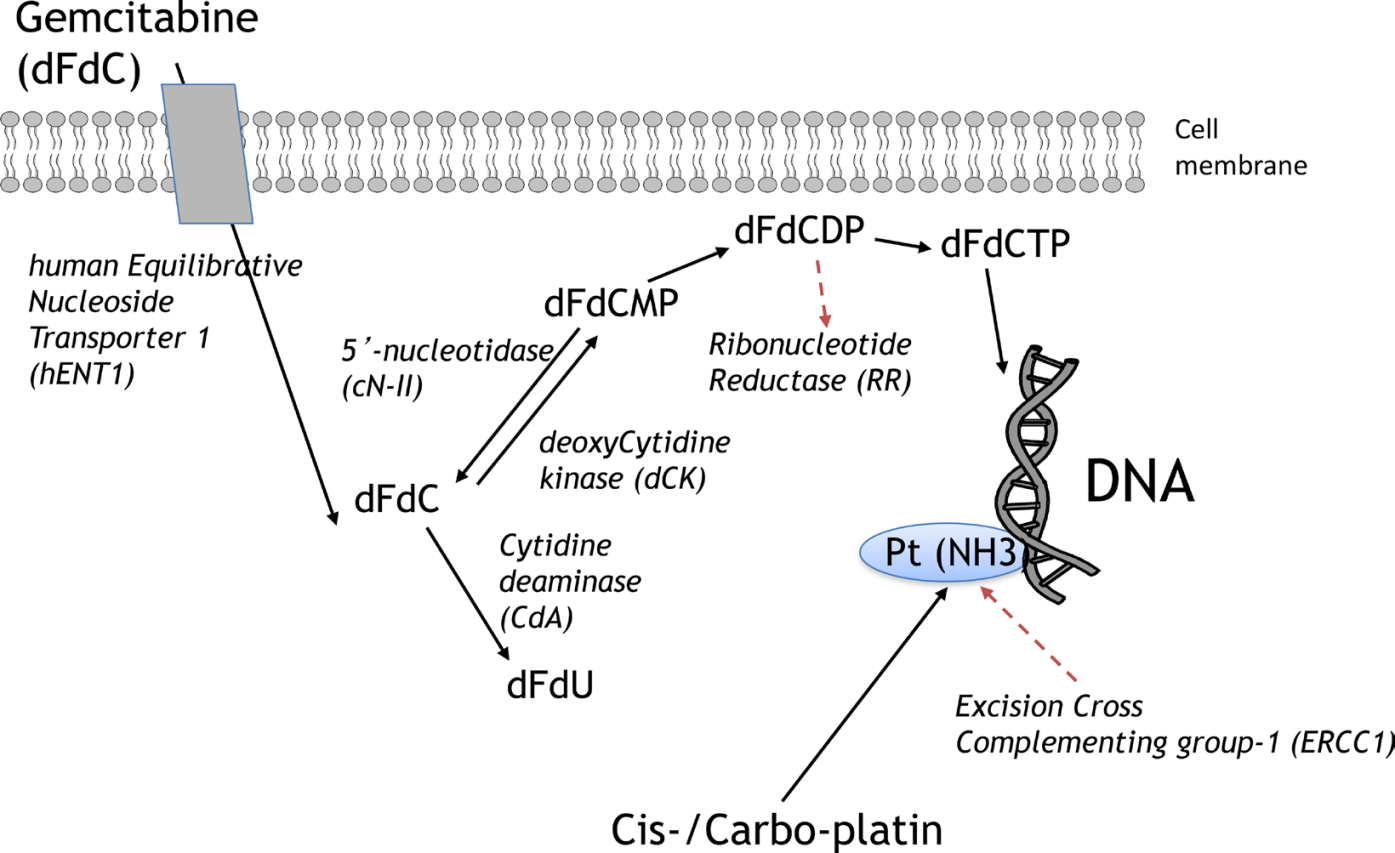
Key determinants of gemcitabine and platinum chemosensitivity/resistance Black lines, metabolism; Red lines, targets of the activity. Abbreviations: dFdCDP, gemcitabine diphosphate; dFdCMP, gemcitabine monophosphate; dFdCTP, gemcitabine triphosphate; dFdU, 2’,2’-difluoro-deoxyuridine.

Based on the above evidence, we evaluated the intratumoral expression of ERCC1, RRM1, RRM2, hENT1, dCK, cN-II and CDA (Figure 1) by validated quantitative-PCR methods in two cohort of NSCLC patients treated with platinum/gemcitabine-based regimens and we correlated gene expression levels with response to treatment and outcome.

## RESULTS

### Treatment efficacy

Fifty-eight patients with locally advanced or advanced disease were treated with a platinum/gemcitabine regimen for at least 2 cycles (Table 1). Before entering the study, the patients of this cohort (named “test cohort”) were subjected to a radiological evaluation by means of a CT scan, which was repeated after 2 or 3 cycles of chemotherapy in order to assess the tumor response rate.

**Table 1:** Patient characteristics of the test and validation cohorts

Characteristics	Test cohort	Validation cohort
**Age at Diagnosis**		
mean ± sd (median)	61.8 ± 8.5 (61.9)	63.5 ± 6.6 (63.1)
min, max	40, 78	50, 78
**Gender, no. (%)**		
Female	17 (29.3)	11 (27.5)
Male	41 (70.7)	29 (72.5)
**Histology, no. (%)**		
NSCLC^*^	27 (46.6)	
SCC	6 (10.3)	17 (42.5)
ADK	24 (41.4)	22 (55.0)
Large Cells	1 (1.7)	1 (2.5)
**Stage, no. (%)**		
IIIA	43 (74.1)	0 (0)
IIIB	7 (12.1)	18 (45.0)
IV	8 (13.8)	22 (55.0)

^*^The morphology was poorly specified and these patients were defined NSCLC.

A radiological response evaluation was possible for all patients enrolled. Of the 58 evaluable patients, one patient (1.7%) had a complete response, 28 (48.3%) showed partial response, 18 (31.1%) had stable disease, while 11 patients (19%) experienced disease progression (Table 2).

**Table 2:** Response to treatment of the patients in the test and validation cohorts

**Chemotherapy cycles**		
mean ± sd (median)	3.4 ± 1.0 (3.0)	4.5 ± 1.3 (4.0)
min, max	2, 6	2, 6
**Responses, no. (%)**		
CR	1 (1.7)	0 (0.0)
PR	28 (48.3)	14 (35.0)
SD	18 (31.0)	20 (50.0)
PD	11 (19.0)	6 (15.0)

Cut-off values for each of the clinical and pathological factors were selected according to the median value for continuous variables, and univariate analysis was carried out to identify those factors significantly associated with outcome. Surprisingly, these analyses showed that those patients obtaining CR/PR were significantly older than patients with SD or PD (*p =* 0.030). Conversely, sex (*p =* 0.387), stage (*p =* 0.390), histology (*p =* 0.692) and chemotherapy (i.e., the chemotherapy regimens included, in addition to gemcitabine, either carboplatin or cisplatin) (*p =* 0.572) were not significantly associated with response.

### Gene expression levels according to response to platinum-gemcitabine therapy

**Figure 2:**
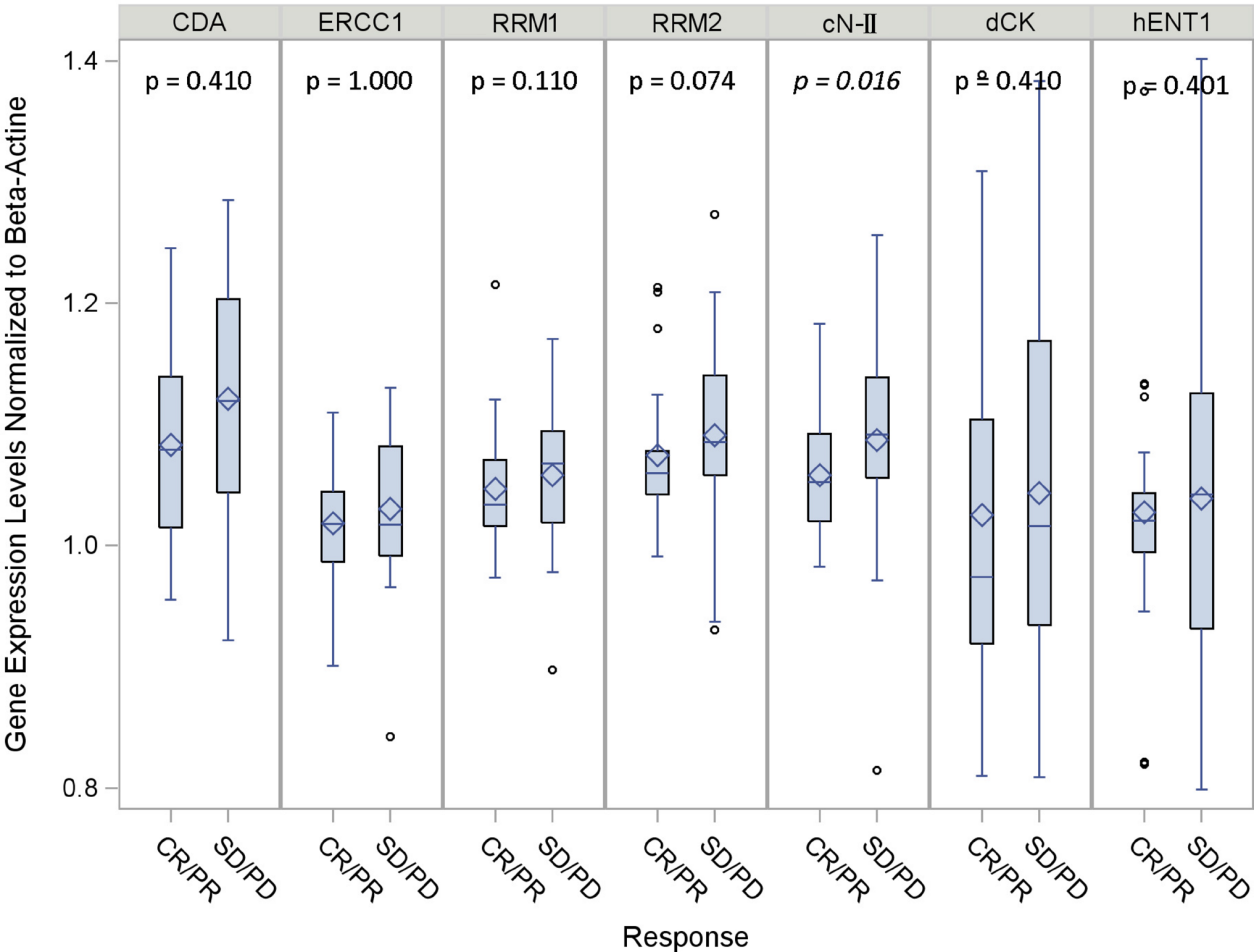
Gene expression levels according to clinical response normalized to β-actin Boxplots distribution of data: the edges of each box indicate the 25th (bottom) and 75th (top) percentiles. The marker and the line inside the box are the mean and the median value respectively. The whiskers indicate values close enough to the box not to be considered outliers. Other points are considered to be outliers.

To find out whether the different gene expression could be correlated to an objective response to platinum-gemcitabine chemotherapy, we evaluated the expression levels of dCK, cN-II, CDA, RRM1, RRM2, hENT1 and ERCC1 by quantitative-PCR, in those patients who achieved a measurable RECIST response (responders, CR or PR) compared to patients who had stable disease or disease progression (non-responders, SD or PD). mRNA levels were normalized to either β-actin or GAPDH. The results showed comparable means and variability in the ratio of each target gene with the two different housekeeping genes (Figure 2 and Supplementary Figure 1).

Expression levels of all the studied genes were not associated with any of the clinical-pathological factors considered above.

The plot in Figure 2 shows the distribution of gene expression levels normalized to β-actin according to tumor response. The observed variability suggests a possible stratification of patients in order to create homogeneous groups with different likelihoods of response to treatment. By adopting cut-off values according to median expression levels, and using β-actin as housekeeping gene, cN-II was the only gene that reached statistical significance for differential expression between the responder and non-responder patients, with lower levels in those patients achieving a better RECIST response (*p =* 0.016; Table 3 and Figure 2). Interestingly all markers but dCK and hENT1 were homogenous in terms of variability, with a CV% ranging from 5.0% (cN-II CR/PR and ERCC1 CR/PR) to 8.1% (CDA overall) (Table 3). The potential use of the median value of cN-II as a predictor of clinical outcome was also suggested by using GAPDH as housekeeping gene, but this was not statistically significant (Supplementary Figure 1).

**Table 3:** Summary statistics of β-actin normalized gene-expression according to response

Marker	Response	No.	Mean ± SD	Median	CV%	*p* (^*^)
**dCK**	Overall	52	1.035 ± 0.020	0.986	14.2	
	CR/PR	25	1.025 ± 0.030	0.974	14.8	0.410
	SD/PD	27	1.044 ± 0.028	1.016	13.8	
**cN-II**	Overall	49	1.072 ± 0.010	1.074	6.7	
	CR/PR	25	1.058 ± 0.011	1.053	5.0	***0.016***
	SD/PD	24	1.087 ± 0.018	1.092	8.0	
**CDA**	Overall	52	1.103 ± 0.012	1.106	8.1	
	CR/PR	25	1.083 ± 0.017	1.079	7.9	0.410
	SD/PD	27	1.121 ± 0.018	1.119	8.1	
**RRM1**	Overall	47	1.053 ± 0.008	1.046	5.4	
	CR/PR	22	1.047 ± 0.012	1.034	5.2	0.110
	SD/PD	25	1.058 ± 0.012	1.068	5.6	
**RRM2**	Overall	44	1.083 ± 0.010	1.062	6.4	
	CR/PR	22	1.075 ± 0.013	1.060	5.5	0.074
	SD/PD	22	1.091 ± 0.017	1.085	7.2	
**hENT1**	Overall	50	1.033 ± 0.018	1.030	12.3	
	CR/PR	25	1.027 ± 0.021	1.021	10.2	0.401
	SD/PD	25	1.039 ± 0.030	1.042	14.2	
**ERCC1**	Overall	50	1.025 ± 0.008	1.017	5.6	
	CR/PR	24	1.018 ± 0.010	1.018	5.0	1.000
	SD/PD	26	1.030 ± 0.012	1.017	6.2	

(^*^) CR/PR vs. SD/PD comparison. Wilcoxon two-sample two-sided test or unpaired *t*-test as appropriate

**Figure 3:**
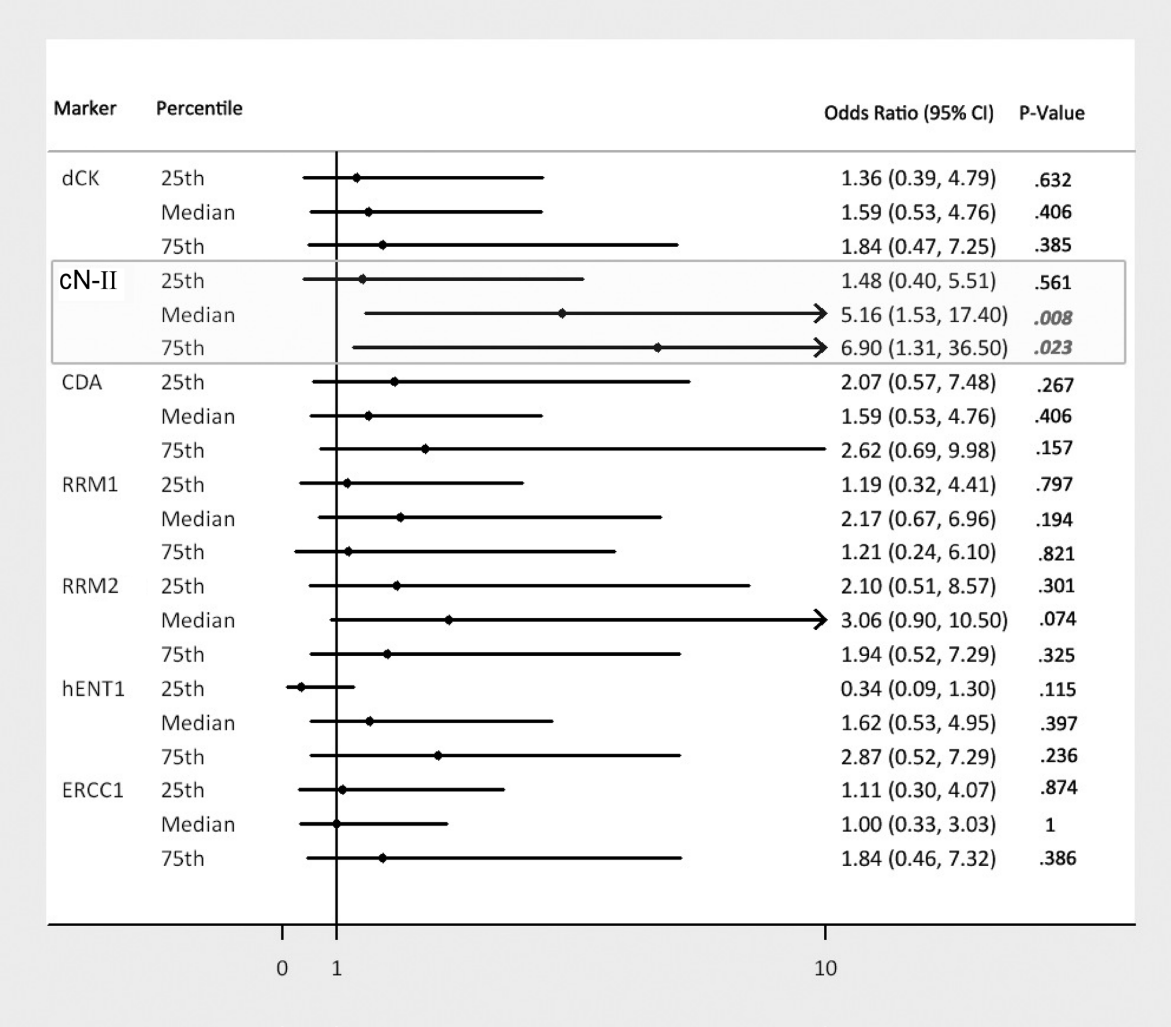
Univariate odds ratios of getting a SD/PD by gene expression marker quantiles A Forest plot type showing the odds-ratios of getting a SD/PD for gene expression marker values greater than a given percentile. The bars represents the 95% confidence intervals around the odds-ratio, the dot is the point estimate of the odds-ratio.

In the univariate logistic regression analysis, we observed a possible “dose-response” relationship between the cN-II expression levels and the risk (odds-ratio) of non-response (Figure 3): the higher the expression level, the higher the odds of non-response. Indeed by using the 25th, 50th (median) and 75th percentiles as cN-II mRNA cut-off levels, we estimated the odd-ratio of 1.48, 5.16 and 6.90 (*p =* 0.56, *p =* 0.08, *p =* 0.023) respectively. Furthermore in the multivariate analysis, cN-II expression still remained a significant predictive factor, with the risk of non-response increasing up to 6.10 times (*p =* 0.047) for cN-II expression levels above median (Table 4).

**Table 4:** Multivariate Odds-ratios of getting a SD/PD according to β-actin normalized gene-expression marker’s median levels (Low vs. High expression)

		Status No. (col %)		
Marker		Odds-Ratio	95% CI	*p* (^*^)
dCK	Low	1		
	High	1.95	0.34, 11.2	0.454
cN-II	Low	**1**		***0.047***
	High	**6.10**	1.02, 36.5	
CDA	Low	**1**		
	High	0.93	0.14, 5.98	0.936
RRM1	Low	**1**		
	High	1.12	0.17, 7.43	0.909
RRM2	Low	**1**		
	High	3.48	0.76, 16.1	0.109
hENT1	Low	**1**		
	High	0.59	0.10, 3.61	0.564
ERCC1	Low	**1**		
	High	0.42	0.08, 2.17	0.298

(^*^) Wald Chi-square test.

### Gene expression levels of cN-II according to survival in the test and validation cohort

**Figure 4:**
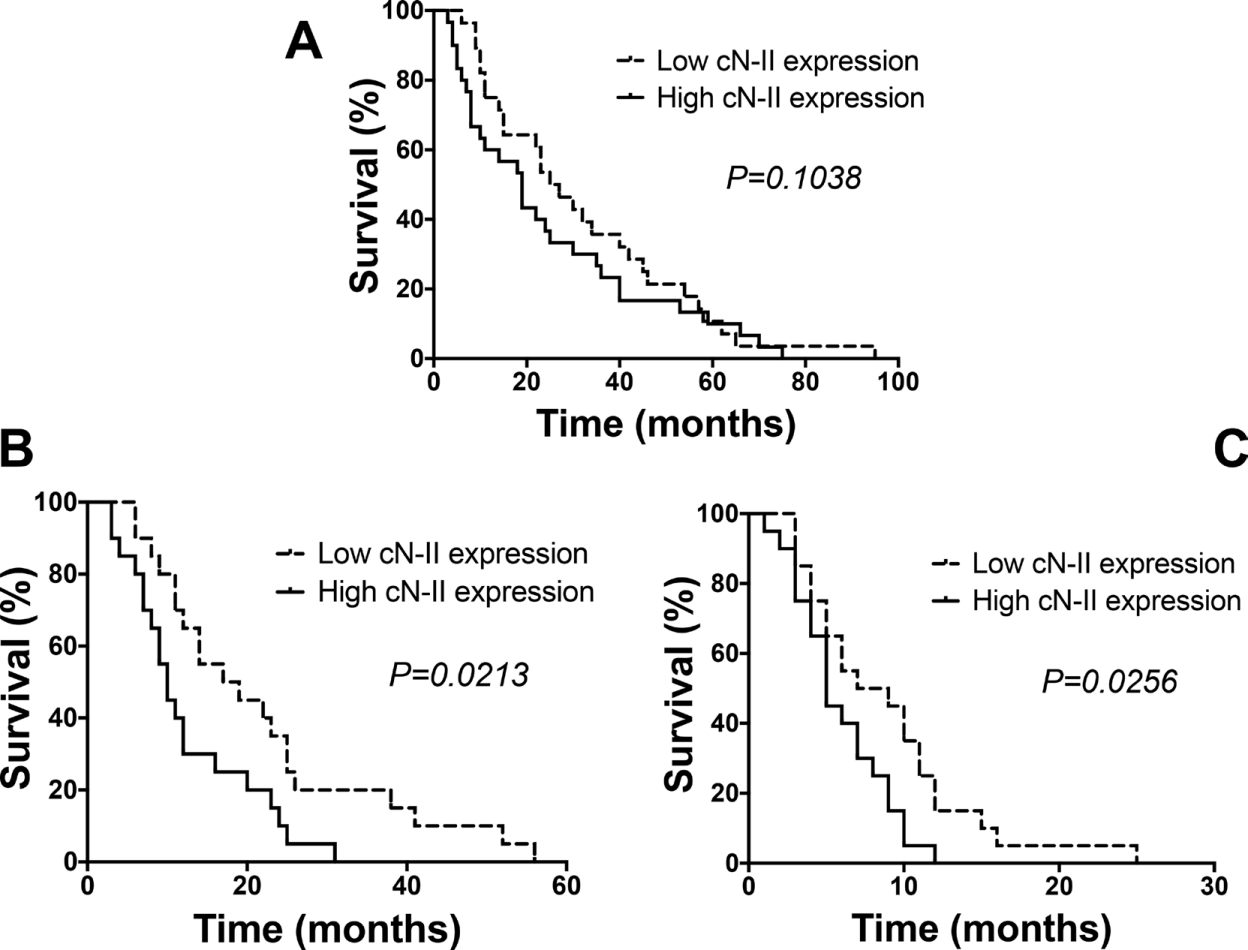
Survival and progression-free survival curves Overall survival (OS) and progression-free survival (PFS) curves segregated according to cN-II mRNA expression levels in the NSCLC patients from the test (**A**) and validation cohort (**B**, **C**). High/low levels are relative to the median values acquired by quantitative-PCR data in laser-microdissected samples available from the patients of the two cohorts. The curves were compared using the log-rank test.

Despite the limited number of patients in advanced stage, we evaluated whether the expression of cN-II correlated with overall survival (OS). As shown in the Figure 4A, patients with “high” expression have an OS of 19.3, compared to 26.5 months of the patients with “low” cN-II expression (*p* = 0.1038). However, we performed further analyses in a validation cohort, including advanced stage patients, treated with gemcitabine-cisplatin, as described previously [32, 33]. The characteristics and response of these patients are described in the Tables 1 and 2. In these patients, by using the median of cN-II mRNA cut-off level, the “high” expression correlated with a significantly increased risk of non-response (*p =* 0.034). Moreover, the progression-free survival (PFS) and OS were significantly longer in patients with “low” cN-II expression (Figure 4B–4C).

### Modulation of gemcitabine antiproliferative effects

**Figure 5:**
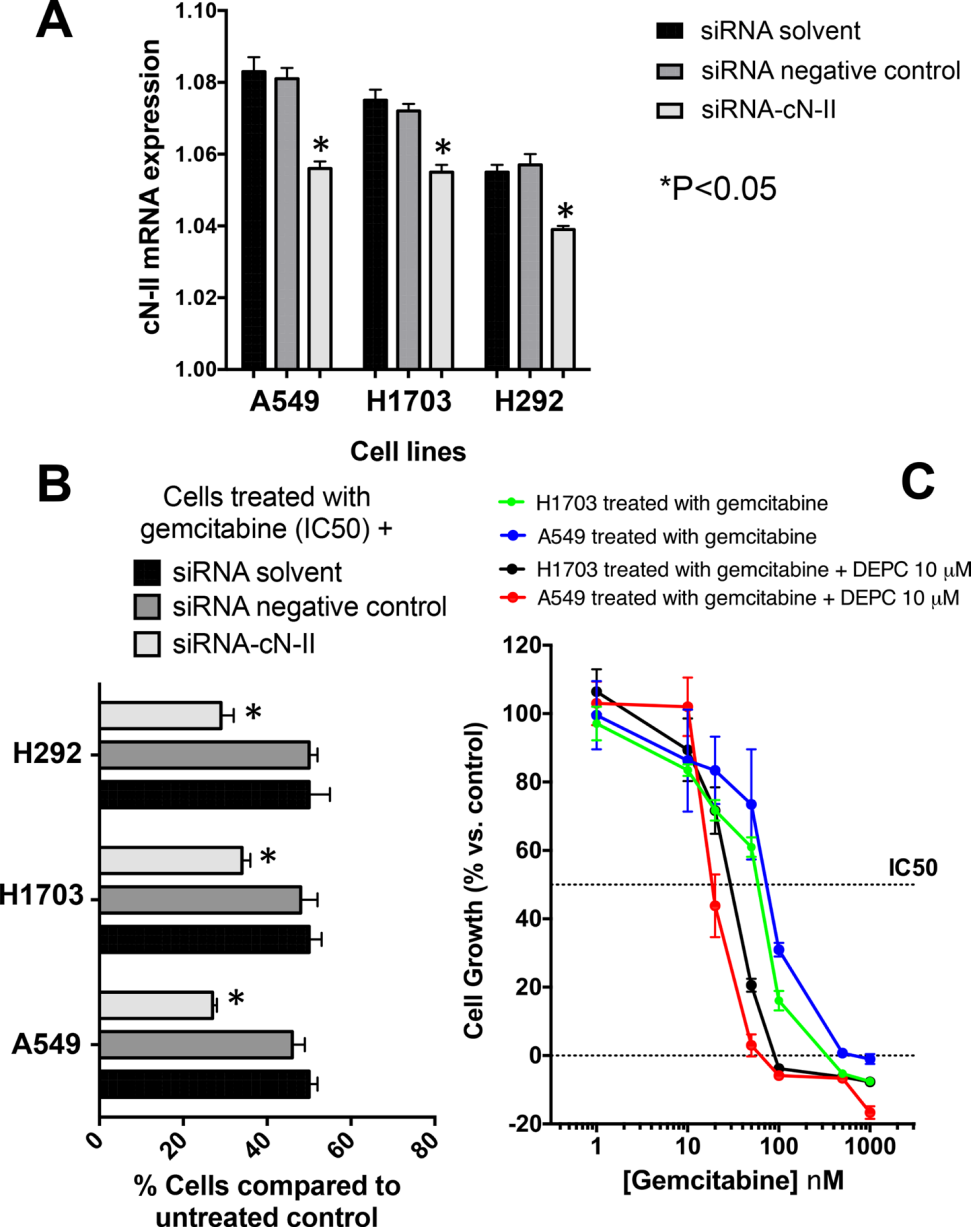
Increased activity of gemcitabine by silencing or pharmacological inhibition of cN-II in NSCLC cell lines (**A**) Quantitative RT-PCR analysis of cN-II mRNA expression in NSCLC cell lines. Results are presented relative to the expression levels of β-actin and show that silencing with a specific siRNA for cN-II (siRNA-cN-II) significantly reduced the expression of cN-II compared to cells treated with the siRNA tranfection reagents, named “siRNA solvent”, in all the three cell lines. (**B**) Cell growth of cells treated with gemcitabine (at the cell line specific IC50) after exposure to siRNA-cN-II. Cell growth of treated cells was compared to growth of untreated control cells set at 100%. (**C**) Representative curves of growth inhibitory effects of gemcitabine and of the simultaneous combination of gemcitabine and of the cN-II inhibitor DEPC in H1703 and A549 cells. The IC50 values were significantly reduced in both cell lines. Similar results were obtained in the H292 cells (data not shown). *Points* and *columns*, mean values obtained from three independent experiments; *bars*, S.E. ^*^Significantly different from (*P* < 0.05) from respective controls (“siRNA solvent”).

The quantitative-PCR evaluation of cN-II in three NSCLC cells showed expression levels similar to those detected in tumor specimens, ranging from 1.083 (A549 cells) to 1.056 (H292 cells). To assess whether the down-regulation of cN-II gene expression similar to the gene expression variability detected in the lung tissues might affect the cytotoxic activity of gemcitabine, we performed specific experiments with siRNA gene silencing against cN-II. The negative-control siRNA did not affect cN-II expression; while we observed a significant reduction of cN-II levels after transfection with 5 nmol siRNA for cN-II (Figure 5A). Importantly, the down-regulation of cN-II was associated with increased sensitivity to gemcitabine, as demonstrated by the significant reduction in the percentages of cell viability (Figure 5B).

Similarly, pharmacological experiments testing the combination with the cN-II inhibitor DEPC showed a decrease of the IC50 values of gemcitabine, as shown by the significantly shifted dose response curves in the A549 and H1703 cells (Figure 5C).

## DISCUSSION

Platinum compounds and gemcitabine are widely used to treat NSCLC patients in different settings of disease and it is of paramount importance to identify predictive biomarkers that could be easily assessed in daily practice to select those patients who can most benefit from these cytotoxic agents. This study evaluated the expression of several key genes involved in the regulation of the activity and metabolism of platinum and gemcitabine in microdissected, frozen NSCLC specimens, and, to our knowledge, is the first to demonstrate a significant association of cN-II mRNA levels with response to platinum and gemcitabine in advanced NSCLC patients.

One main strength of this study is that gene expression was assessed by a validated PCR method in frozen specimens, that were laser-microdissected to avoid the contamination of normal cells surrounding the tumor. Specimens were obtained by various methods and from various sites, including metastatic lymph nodes. However, in our previous study in 88 laser-microdissected samples from NSCLC patients we did not observe a significant difference between primary tumor and tumor metastasis, supporting the use of both lymph nodes and primary tumors for the expression profiling of NSCLC [14].

Moreover, we used two different housekeeping genes, GAPDH and β-actin, to improve the reliability of the results. Interestingly, the pattern of gene expression was comparable with both the housekeeping genes, even if the role of cN-II as a predictor of platinum-gemcitabine efficacy showed only a trend toward statistically significant difference, with GAPDH. However, the utility of GAPDH as a good reference sequence has been criticized since human genome contains many GAPDH pseudogenes which have identical or nearly identical sequences to the active, target GAPDH transcript, and therefore primers or probes spanning exon junctions will detect the presence of the pseudogenes along with the cDNA of the active transcript [34].

The main objective of this study was to assess the correlation of gene expression with tumor response. Indeed, RECIST tumor evaluation gives objective information on drug activity, without being influenced by other variables, including further treatment lines, which can instead influence overall survival. Furthermore, the identification of genes that could predict a reduction in tumor size could be particularly relevant in the neoadjuvant setting, where platinum/gemcitabine regimen is also widely used. We observed an inverse correlation between cN-II mRNA levels and response to a platinum-gemcitabine treatment, as detected using the 25th, 50th (median) and 75th percentiles as cut-off levels, with lower cN-II expression associated to a better RECIST response. Its role as a predictor of platinum-gemcitabine efficacy was confirmed also in multivariate analysis, with a risk of non-response increased up to 6 times for cN-II levels above median. Despite the patient number of advanced stage was very small, we also observed a trend toward increased survival in patients with low cN-II expression. However, we observed a significant correlation between cN-II expression and both PFS and OS in an independent validation cohort of stage IIIB/IV patients.

This cytoplasmic nucleotidase is a key enzyme involved in gemcitabine inactivation, but can also affect the metabolism and activity of other nucleoside analogues, including fludarabine, cytarabine and cladribine. Several studies have correlated the expression of cN-II to clinical outcome to treatment with nucleoside analogues in patients with hematological malignancies and solid tumors [35]. A high cN-II activity was observed in resistant chronic lymphocytic leukaemia patients treated with cladribine [36], as well as in human acute T lymphoblastic leukaemia cells [30]. Regarding NSCLC, Sève *et al.* assessed the protein abundance of cN-II, hENT1, the human concentrative nucleoside transporter-3 (hCNT3) and dCK by immunohistochemistry (IHC), in tumors of patients with locally or advanced disease, treated with gemcitabine, and correlated it with clinical outcome [23]. Among the 43 samples analyzed, 36 (86%) expressed cN-II and this was the only protein associated with overall survival, while no association was found with response. This apparent difference with our results could be due to the different methods used for measuring cN-II levels. Indeed, no information is available about the correlation between cN-II mRNA levels, as measured by quantitative-PCR, and protein expression detected by IHC. In order to correlate cN-II protein expression with tumor reduction, we performed cN-II IHC analysis as well. Unfortunately, cN-II staining (monoclonal antibody against cN-II clone ECNII) was not successful in our study samples (data not shown).

To evaluate whether modulation of gene expression similar to the gene expression variability detected in the lung tissues might affect the cytotoxic activity of gemcitabine, we performed specific experiments with siRNA gene silencing against cN-II in three NSCLC cell lines. In these experiments, we observed expression differences comparable with those detected in the clinical samples, and cytotoxicity assays showed that down-regulation of cN-II caused a significant increase of gemcitabine sensitivity. As negative-control siRNA, which did not affect cN-II expression, did not alter the cell growth, these results indicate that the modulation of gemcitabine cytotoxicity was determined by the specific perturbation of the expression of cN-II. Further experiments showed a significant decrease of the IC50 values of gemcitabine after simultaneous treatment with gemcitabine and the cN-II inhibitor DEPC. These results are in agreement with previous studies showing increased sensitivity to gemcitabine after treatment with DEPC in pancreatic, lymphoid and lung cancer cell lines [37–39]. We can therefore hypothesize that this knowledge could be translated also for other tumor types where gemcitabine alone or the combination of gemcitabine/cisplatin is used, such as cholangiocarcinomas or urinary tract cancers [40, 41].

It is well recognized that the presence of single nucleotide polymorphisms (SNPs) in crucial genes involved in drug metabolism can influence the expression or functions of corresponding proteins, thus contributing to inter-individual variability in response and clinical outcome to specific classes of cytotoxic drugs. Indeed, recent studies in lymphoblastoid cell lines with the common polymorphism rs2274341 in the cN-II had lower cN-II expression and cytarabine sensitivity [42] and the presence of this polymorphism in lung cancer patients treated with gemcitabine was significantly associated with a better survival [42, 43]. We can therefore hypothesize that the presence of single nucleotide polymorphisms (SNPs) of the cN-II gene might affect also clinical response by modulating cN-II expression, and further studies on these SNPs are warranted.

The expression of cN-II might be influenced by several other factors. In particular, some recent studies suggested the key role of microRNA (miRNA) in the regulation on key determinants of chemotherapeutic drugs activity [44]. According to the miRTarBase database a total of 62 miRNAs might target cN-II, but only 6 miRNAs were validated in next-generation sequencing studies in more than two articles, as described in the Supplementary Table 1.

We also assessed RRM1 and ERCC1, whose high expression has been shown to correlate with resistance in advanced NSCLC patients treated with gemcitabine and platinum in a number of retrospective studies [24–27, 17, 18]. However, no prospective trials have validated their role in customizing systemic therapy [45, 46, 17]. Also, in our study we did not find any correlation between ERCC1 or RRM1 mRNA expression and clinical response to treatment.

These results are limited by the small sample size in each cohort. Two other important limitations of the present study are that the *p* values were not corrected for multiple hypotheses, and that the significant correlation of cN-II levels was demonstrated under very specific conditions, namely, by use of the median as a cut off and with normalization of mRNA expression levels according to only one housekeeping expression pattern.

In conclusion, cN-II expression emerged as the only predictor of responsiveness to treatment with gemcitabine and platinum. These results suggest a potential prognostic/predictive value of cN-II and should prompt further validation in prospective studies in larger cohorts of patients, that will be crucial to demonstrate whether cN-II could be useful in the clinical setting to personalize chemotherapy in NSCLC patients.

## MATERIALS AND METHODS

### Patient characteristics

From 2004 to 2008, a total of 58 chemotherapy-naive patients affected by locally advanced or metastatic NSCLC were treated with a platinum/gemcitabine-based combination regimen at the European Institute of Oncology and were enrolled in our study. Characteristics of patients are shown in Table 1. In summary, there were 17 women and 41 men, with age at diagnosis ranging from 40 to 78 years. Forty-three patients had stage IIIA disease, 7 stage IIIB and 8 stage IV disease.

Before initiating neo-adjuvant or first-line chemotherapy, patients were subjected to tumor biopsy, including bronchoschopy (1/58), mediastinoscopy (41/58), minithoracotomy (2/58), supraclavicular lymph node biopsy (7/58), video-assisted thoracoscopy (5/58) or liver biopsy (2/58) with collection of fresh tumor specimens. Gene expression analyses were performed in one (1.7%) sample from the primary tumor, 50 (86.2%) from metastatic lymph nodes and 7 (12.1%) from distant metastases.

All the patients received a standard platinum-based treatment (46 patients received cisplatin and 12 carboplatin) in association with gemcitabine, for at least 2 cycles. Assessment of tumor response was carried out by computed tomography (CT) scan every two or three cycles. Responses were assessed by using Response Evaluation Criteria in Solid Tumors (RECIST). [47].

All patients gave written informed consent. The study was approved by the local Institutional Ethics Committees and was conducted in accordance with Good Clinical Practice guidelines and the Declaration of Helsinki.

### Independent validation cohort

Further studies on the correlation of the expression of cN-II with clinical outcome were performed in laser-microdissected specimens from an independent validation cohort of 40 chemotherapy-naïve patients with histologically or cytologically proven NSCLC and measurable clinical stage IIIB or IV disease from Livorno Civil-Hospital (Livorno, Italy). Eighteen patients had stage IIIB and 22 stage IV disease. All these patients were subjected to tumor biopsy, and then received chemotherapy, which consisted of cisplatin 80 mg/m^2^ infused over 60 minutes on day-1 and gemcitabine 1,200 mg/m^2^ administered intravenously over 30 minutes on day-1 and 8, every 3 weeks for a maximum of 6 courses. Treatment was discontinued in case of progression, major toxicities, or according to the patient’s or physician’s decision.

### Sample collection and processing

Frozen tissue sections (5 μm) were thawed, fixed in 75% ethanol, and dehydratated in 100% ethanol and xylene. Neoplastic cells were then dissected using the laser microdissector Leica AS/LMD (Leica, Wetzlar, Germany). Laser-captured cells were pooled in lysis buffer and RNA was extracted with the QIAamp RNA Mini kit (Qiagen, San Diego, CA, USA). RNA was dissolved in DNase/RNase-free water, and measured by absorbance reading at 260/280 nm. RNA yields and integrity were checked at 260–280nm with NanoDrop^®^-1000-Detector (NanoDrop-Technologies, USA).

### Quantitative-PCR analysis

From 50 to 500 ng RNA was reverse-transcribed. The resulting cDNA was amplified with the 7900HT sequence detection system (Applied Biosystems, Foster City, CA, USA). Forward and reverse primers and probes were designed with Primer Express 2.0 (Applied Biosystems) based on dCK (NM_000788), cN-II (NM_012229), and CDA (NM_001785) gene sequence obtained from the GenBank, whereas primers and probes for RRM1 (NM_001033), RRM2 (NM_001034), hENT1 (NM_004955) and ERCC1 were obtained from Applied Biosystems Assay-on-Demand products (Hs00168784, Hs0035724, Hs00191940 and Hs01012158). Validation experiments were carried out with cDNA obtained from QPCR Human Reference Total RNA (Stratagene, La Jolla, CA, USA), as described previously [48, 49]. Specimens were amplified in triplicate with appropriate nontemplate controls, and the coefficient of variation was <2% for all replicates.

### Effects of inhibition of cN-II on gemcitabine cytotoxicity

To investigate whether the gene expression variability of cN-II observed between tumor tissues might affect the cytotoxic activity of gemcitabine, we evaluated the effects of a specific siRNA (Assay ID#36451, ThermoFisher Scientific, Waltham, MA, USA) against cN-II in three NSCLC cell lines (A549, H292 and H1703) characterized by high, intermediate and low cN-II mRNA levels, as assessed by quantitative-PCR.

Cells were plated at 10^5^ cells per well in 6-well plates and, after 24 hours, were transfected with small interfering RNA (siRNA) oligonucleotide or negative-control siRNA using Oligofectamine (Invitrogen) to result in a final RNA concentration of 5 and 25 nmol/l in serum-free medium, according to the manufacturer’s instructions. After 24 hours, the cells were treated with gemcitabine (0.01–500 nM) for 48 hours, while RNA was extracted from parallel wells, using the TRI REAGENT LS (Sigma-Aldrich). At the end of drug treatment, the cell growth inhibitory effect of gemcitabine was studied by direct cell count using the trypan blue. Growth inhibition was expressed as the percentage of gemcitabine-untreated controls (untransfected and negative-control siRNA-treated cells), and the 50% inhibitory concentration of cell growth (IC50) was calculated by non-linear least squares curve fitting (GraphPad PRISM, Intuitive Software for Science, San Diego, CA, USA).

Additional pharmacological studies were performed with the cN-II inhibitor diethylpyrocarbonate (DEPC). Cells were plated in 96-well plates, and treated with gemcitabine for 72 hours alone or in combination with 10 μM DEPC. The cell growth inhibitory effects were studied using the 3-(4,5-dimethylthiazol-2-yl)-2,5-diphenyltetrazolium (MTT) assay. IC50 values were calculated as described above.

### Statistical analyses

Demographic and clinical information were obtained from medical records. Summary statistics (n, mean, standard errors, median and CV%) for all gene expression normalized either to GAPDH or β-actin by clinical response, as well as by pathological response and overall survival (OS) were either tabulated or box-plotted. Factors included in the univariate analysis were sex, age at diagnosis, clinical stage and histology.

Comparison between gene expression levels and clinical response (complete response, CR, or partial response, PR, defined according to RECIST criteria), as well as for stable disease (SD) + progressive disease (PD) and other class-levels of clinico-pathological factors were performed using Fisher’s exact two-sided test, Kruskal–Wallis test, median test or unpaired Students *t*-test as appropriate (Satterthwaite method was adopted for unequal variances as tested by the Folded F method). Normality was tested using the Shapiro–Wilk test.

Univariate and multivariate logistic regressions were carried out to determine the association between gene expression and therapy responsiveness, calculating the crude and adjusted odds ratio (OR) with 95% confidence intervals (CI) using the first, second and third quartiles of the gene expression distributions. Lower quartiles were used as reference category. The level of significance was set at 5%, all test were two-sided. The Kaplan–Meier method was used to plot overall survival, and the log-rank test was used to compare curves in univariate analysis. Data were analyzed using SAS 9.2 (Cary, NC, USA) and STATA/SE 11.1 (StataCorp., TX, USA).

All *in vitro* experiments were performed in triplicate and were repeated at least three times. Data were expressed as mean values S.E. and were analyzed by Student’s *t* test or analysis of variance followed by the Tukey’s multiple comparisons; the level of significance was set at *P < 0.05*.

## SUPPLEMENTARY MATERIALS FIGURES AND TABLES




