# Supplementary material for: 5'-nucleotidase cN-II emerges as a new predictive biomarker of response to gemcitabine/platinum combination chemotherapy in non-small cell lung cancer
Source: Oncotarget. 2018 Feb 16;9(23):16437–50. doi: 10.18632/oncotarget.24505 (PMC5893252; doi:10.18632/oncotarget.24505)
Supplement: Supplementary file 1 [file oncotarget-09-16437-s001.pdf]

5'-nucleotidase cN-II emerges as a new predictive biomarker of response to gemcitabine/platinum combination chemotherapy in non-small cell lung cancer

SUPPLEMENTARY MATERIALS

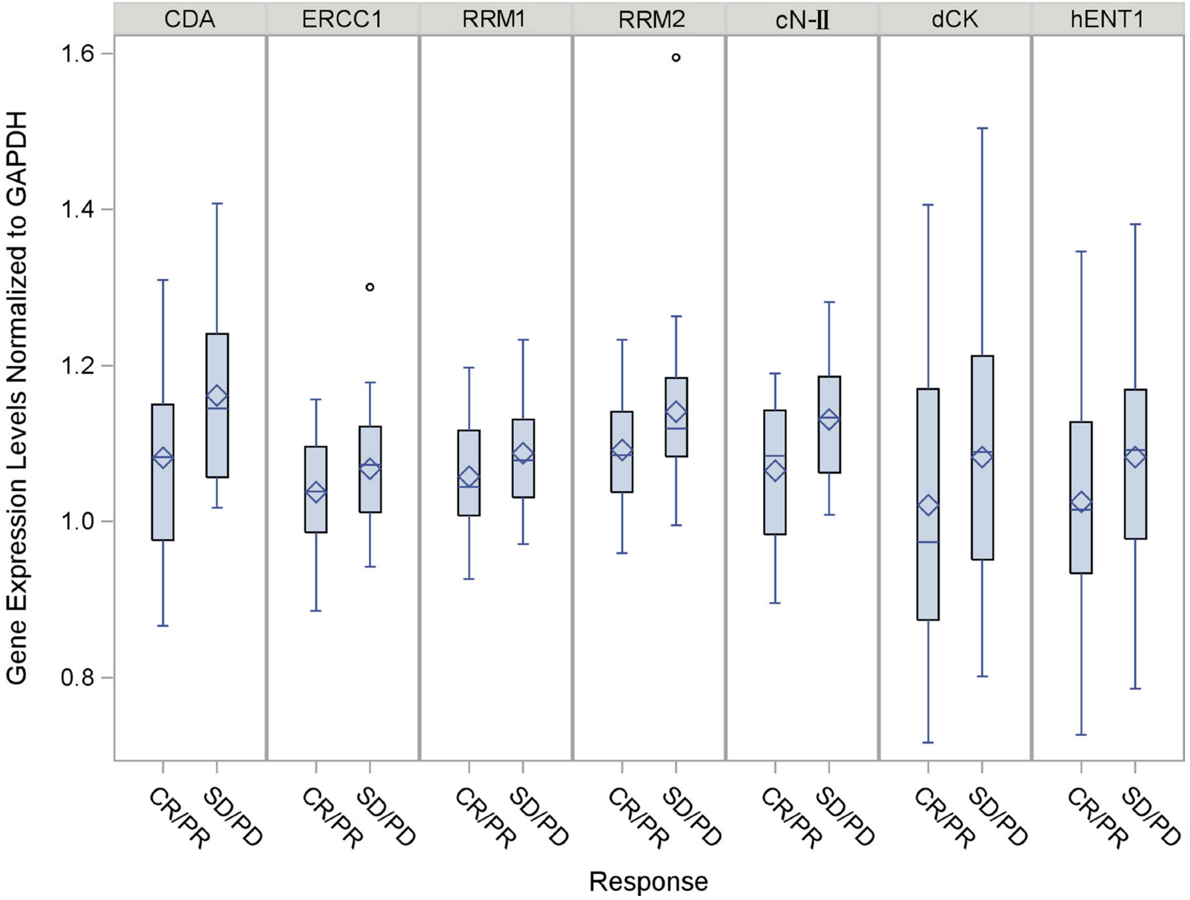

Supplementary Figure 1: Gene expression levels according to clinical response normalized to GAPDH. Boxplots distribution of data: the edges of each box indicate the 25th (bottom) and 75th (top) percentiles. The marker and the line inside the box are the mean and the median value respectively. The whiskers indicate values close enough to the box not to be considered outliers. Other points are considered to be outliers.

Supplementary Table 1: MiRNA targeting cN-II, as reported in *miRTarBase* as validated by NGS (Release 7.0: Sept. 15, 2017). See Supplementary_Table_1
