# Supplementary material for: 5'-nucleotidase cN-II emerges as a new predictive biomarker of response to gemcitabine/platinum combination chemotherapy in non-small cell lung cancer
Source: Oncotarget. 2018 Feb 16;9(23):16437–50. doi: 10.18632/oncotarget.24505 (PMC5893252; doi:10.18632/oncotarget.24505)
Supplement: Supplementary file 2 [file oncotarget-09-16437-s002.docx]

**Supplementary Table 1: MiRNA targeting cN-II, as reported in**

***miRTarBase* as validated by NGS (Release 7.0: Sept. 15, 2017)**

| MiRNA targeting cN-II | Number of articles |
| --- | --- |
| hsa-miR-214-5p | 2 |
| hsa-miR-5089-5p | 2 |
| hsa-miR-550a-5p | 2 |
| hsa-miR-550a-3-5p | 2 |
| hsa-miR-1271-3p | 2 |
| hsa-miR-619-5p | 2 |
| hsa-miR-4722-3p | 1 |
| hsa-miR-544b | 1 |
| hsa-miR-4324 | 1 |
| hsa-miR-660-3p | 1 |
| hsa-miR-5571-5p | 1 |
| hsa-miR-498 | 1 |
| hsa-miR-23b-5p | 1 |
| hsa-miR-23a-5p | 1 |
| hsa-miR-550b-2-5p | 1 |
| hsa-miR-6514-3p | 1 |
| hsa-miR-6894-3p | 1 |
| hsa-miR-6811-3p | 1 |
| hsa-miR-6889-3p | 1 |
| hsa-miR-1914-5p | 1 |
| hsa-miR-6752-3p | 1 |
| hsa-miR-6729-3p | 1 |
| hsa-miR-6848-3p | 1 |
| hsa-miR-6843-3p | 1 |
| hsa-miR-6131 | 1 |
| hsa-miR-4454 | 1 |
| hsa-miR-3663-5p | 1 |
| hsa-miR-5699-3p | 1 |
| hsa-miR-4421 | 1 |
| hsa-miR-4485-5p | 1 |
| hsa-miR-3934-5p | 1 |
| hsa-miR-764 | 1 |
| hsa-miR-125a-3p | 1 |
| hsa-miR-3148 | 1 |
| hsa-miR-4477b | 1 |
| hsa-miR-375 | 1 |
| hsa-miR-34a-5p | 1 |
| hsa-miR-21-5p | 1 |
| hsa-miR-193b-3p | 1 |
| hsa-miR-7151-3p | 1 |
| hsa-miR-5095 | 1 |
| hsa-miR-661 | 1 |
| hsa-miR-3116 | 1 |
| hsa-miR-4537 | 1 |
| hsa-miR-1254 | 1 |
| hsa-miR-6506-5p | 1 |
| hsa-miR-3922-3p | 1 |
| hsa-miR-3176 | 1 |
| hsa-miR-6504-3p | 1 |
| hsa-miR-6746-3p | 1 |
| hsa-miR-4438 | 1 |
| hsa-miR-6869-5p | 1 |
| hsa-miR-4279 | 1 |
| hsa-miR-1200 | 1 |
| hsa-miR-6829-3p | 1 |
| hsa-miR-6791-3p | 1 |
| hsa-miR-1976 | 1 |
| hsa-miR-3653-5p | 1 |
| hsa-miR-6747-3p | 1 |
| hsa-miR-6727-3p | 1 |
| hsa-miR-651-3p | 1 |
| hsa-miR-7856-5p | 1 |

From: http://mirtarbase.mbc.nctu.edu.tw/php/index.php

*miRTarBase* is an experimentally validated microRNA-target interactions database, which has accumulated more than three hundred and sixty thousand miRNA-target interactions (MTIs), which are collected by manually surveying pertinent literature after NLP of the text systematically to filter research articles related to functional studies of miRNAs. Generally, the collected MTIs are validated experimentally by reporter assay, western blot, microarray and next-generation sequencing experiments. While containing the largest amount of validated MTIs, the miRTarBase provides the most updated collection by comparing also with other similar, previously developed databases.

Reference:

Chou CH, Chang NW, Shrestha S, Hsu SD, Lin YL, Lee WH, Yang CD, Hong HC, Wei TY, Tu SJ, Tsai TR, Ho SY, Jian TY, et al. miRTarBase 2016: updates to the experimentally validated miRNA-target interactions database. Nucleic Acids Res. 2016; 44:D239-47.
